# Editorial

**Published:** 1898-10

**Authors:** 


### Editorial.

### CHRISTIAN SCIENCE IN ATLANTA.

Owing to the social prominence of the leaders in this special type of religio-medical heterodoxy, Christian Science has had a strong following in Atlanta. Its devotees have maintained a place of meeting and are now on the point of erecting a church. For ten years they have practiced their befogged nonsense for money in open violation of the law.

In the death of one of their eternal gullibles of typhoid fever a short time since, it is hoped the cult will be dealt a heavy blow and many neophytes be frightened off.

A woman living on Courtland street was attacked by typhoid fever. Christian Scientists were summoned and for three days they "wrastled" in prayer, at so much per "wrastle," for salvation of the body, which, unlike that of the soul, is not free.

But prayers and incantations were unavailing, and those recalcitrant little skeptics—the baccilli of Eberth—they only laughed. At length a doctor was called in, but science had done its allotted task,—real science, not luminous decay—the science that teaches unless disease be antagonized the organism invaded must die,—and so the soul, that *quasi* science strove to keep in its "non-existent" tabernacle, passed to its reward.

A reporter from an afternoon paper was sent to interview the "wrastler" in chief. The following is quoted from his interview:

Mrs. Downs was seen at her home this morning and asked. what was the direct cause of the death of Mrs. Lindsay At first she was not inclined to talk, and said that the newspapers had no right to pry into the affairs of people, but finally said:

"It was a combination of mortal thought and chemicals that killed Mrs. Lindsay. It was that and nothing but that.

"The neighbors around the Lindsay home are ignorant and they clamored for a physician and one was called in. It was an effort to appease mortal thought that did this.

"The confusion caused by mortal thought and the absence of strength given by the Christ faith was more than Mrs.

Lindsay was able to resist, and she died. When the physician was called in I left the house and have not been there since.

"Mrs. Lindsay was doing beautifully while under the Christ cure. She did not want a physician, neither did her husband. They called one in because of the clamor made by the neighbors.

"Now it was published that while we had charge of the case that we would not even give Mrs Lindsay ice-water. Such a statement," continued Mrs. Downs, pronouncing the letters and not the word, "is a l-i-e, and I can not see why it should have been printed.

"Why!" exclaimed Mrs. Downs, 'we are not brutes. We are the most humane people in the world. The human mind and mortal thought are not humane, and we are. Mrs. Lindsay had ice-water, and plenty too, but she did not want it.

"There is no necessity," said Mrs. Downs, "to say anything about the Christian Scientists having any connection with the case, as we left when the physician came in."

When asked if Christian Science healers made any charge for their services, Mrs. Downs said: "Certainly we do. Christ said the laborer was worthy of his hire, and we couldn't keep up our rooms unless we accepted pay. We never send in a bill like a physician, but it is generally understood that we are obliged to have money."

Mrs. Lindsay will be buried this afternoon at Hollywood. The husband says: "I am just as firm in my belief as ever."

Comment is well nigh superfluous. The cause of death, "mortal thought and chemicals," scarcely measures up to even the elastic requirements of a death certificate. Perhaps a little more "mortal thought," if that be another mode of expressing common sense, combined with the judicious and timely use of "chemicals," might have averted the calamity. Mortal thought as a cause of death will not figure largely in the mortuary returns of the Christian Scientists. They are as a class immune to the operations of common sense.

As for "chemicals" an occasional brisk purge with calomel would help them to a belief in the existence of matter and at the same time dispel much of that religious fervor which comes from sluggish peristalsis and hepatic torpor.

Hid behind this farce is tragedy. Human lives are jeopardized and even lost. It would be well for the law makers and supporters to think of this.

So long as the whims and vagaries of hysterical women and flabby men are the objects of this "Christ cure," it can do no harm and may be considered as a silly diversion for old maids and childless married women, with such hen-pecked specimens of masculinity as they can bring into the fold. But when a serious disease is concerned—one which endangers life or menaces its security; one which requires skilled medical attention and prompt and judicious treatment—it is no longer a joke. The insane bigots should be caught by the firm hand of the law and thrust *sans ceremonie* out of doors.

If death occur under this charlatanry it is manslaughter, as has been adjudged in other States. Then why not in this?

Is human life so mean a thing that it is allowed to slip away with no one to hold out a staying hand? All good and pious people believe in the efficicacy of prayer. At the same time they have a firm faith in the power of drugs. The mother prays over her sick little one and arises from her knees to follow the doctor's directions. She believes that it is God who gives skill to the physician, and in the good effect of the medicine she sees the answer to her prayer. This is not very scientific. But it is comforting and Christian and true.

Who would surrender this wholesome belief for the inchoate nonsense of a lot of ignorant women and eunuchs? None but plastic weaklings. But these, like the poor, we have always with us.

---

Dr. John M. Langsdale has retired from the editorship of *Langsdale's Lancet*, to be succeeded by Dr. John Punton. The publication will be known hereafter as the *Kansas City Lancet*. Dr. Langsdale built up an exceedingly attractive journal and his retirement is to be regretted.

---

It will be a great matter of gratification to the friends of the coalition to learn that the Atlanta College of Physicians and Surgeons opened its first session with 145 matriculates. Before the end of the first half the number will probably be increased to 250. The greatest harmony prevails and there is every augury of success and prosperity.

Dr. Donald Taylor, acting assistant surgeon, son of Maj. B. D. Taylor, died at Fort McPherson, October 16th, of peritonitis from perforation during the course of an attack of typhoid fever.

L. Stoeber, a second-year medical student and member of the hospital corps, died at Fort McPherson of the same cause. His death was peculiarly sad. He had taught himself the rudiments of an education, and since his matriculation in the then Southern Medical College two years ago, he had given evidence of ability and was a most diligent and indefatigable student. It is unfortunate that his heroic progress toward the end of his ambition should have been untimely cut short.

---

The Kentucky School of Medicine Controversy has been decided permanently by the Court of Appeals in favor of Drs. Kelley and Woody, Dr. Wathen being enjoined from claiming to be dean of the school.

---

The *Atlantic Weekly* suspended publication on October 1st. The unexpired subscriptions have been transferred to the *Philadelphia Medical Journal*.

